# The 380 kb pCMU01 Plasmid Encodes Chloromethane Utilization Genes and Redundant Genes for Vitamin B_12_- and Tetrahydrofolate-Dependent Chloromethane Metabolism in *Methylobacterium extorquens* CM4: A Proteomic and Bioinformatics Study

**DOI:** 10.1371/journal.pone.0056598

**Published:** 2013-04-09

**Authors:** Sandro Roselli, Thierry Nadalig, Stéphane Vuilleumier, Françoise Bringel

**Affiliations:** Département Génétique Moléculaire, Génomique, Microbiologie, Université de Strasbourg, UMR7156, Centre national de la recherche scientifique, Strasbourg, France; Belgian Nuclear Research Centre SCK/CEN, Belgium

## Abstract

Chloromethane (CH_3_Cl) is the most abundant volatile halocarbon in the atmosphere and contributes to the destruction of stratospheric ozone. The only known pathway for bacterial chloromethane utilization (*cmu*) was characterized in *Methylobacterium extorquens* CM4, a methylotrophic bacterium able to utilize compounds without carbon-carbon bonds such as methanol and chloromethane as the sole carbon source for growth. Previous work demonstrated that tetrahydrofolate and vitamin B_12_ are essential cofactors of *cmuA*- and *cmuB*-encoded methyltransferases of chloromethane dehalogenase, and that the pathway for chloromethane utilization is distinct from that for methanol. This work reports genomic and proteomic data demonstrating that cognate *cmu* genes are located on the 380 kb pCMU01 plasmid, which drives the previously defined pathway for tetrahydrofolate-mediated chloromethane dehalogenation. Comparison of complete genome sequences of strain CM4 and that of four other *M. extorquens* strains unable to grow with chloromethane showed that plasmid pCMU01 harbors unique genes without homologs in the compared genomes (*bluB2*, *btuB*, *cobA*, *cbiD*), as well as 13 duplicated genes with homologs of chromosome-borne genes involved in vitamin B_12_-associated biosynthesis and transport, or in tetrahydrofolate-dependent metabolism (*folC2*). In addition, the presence of both chromosomal and plasmid-borne genes for corrinoid salvaging pathways may ensure corrinoid coenzyme supply in challenging environments. Proteomes of *M. extorquens* CM4 grown with one-carbon substrates chloromethane and methanol were compared. Of the 49 proteins with differential abundance identified, only five (CmuA, CmuB, PurU, CobH2 and a PaaE-like uncharacterized putative oxidoreductase) are encoded by the pCMU01 plasmid. The mainly chromosome-encoded response to chloromethane involves gene clusters associated with oxidative stress, production of reducing equivalents (PntAA, Nuo complex), conversion of tetrahydrofolate-bound one-carbon units, and central metabolism. The mosaic organization of plasmid pCMU01 and the clustering of genes coding for dehalogenase enzymes and for biosynthesis of associated cofactors suggests a history of gene acquisition related to chloromethane utilization.

## Introduction

**Figure 1:**
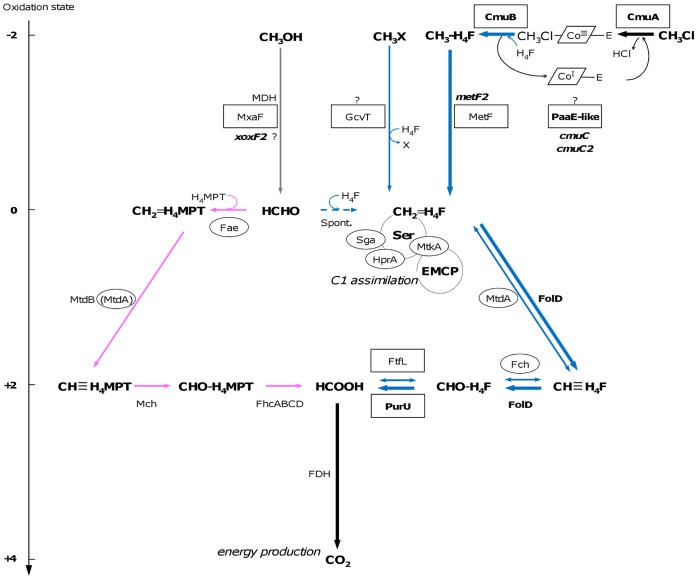
Methylotrophic metabolism and chloromethane utilization pathway in ***Methylobacterium extorquens*** **CM4.** The left-hand scale indicates carbon oxidation state. The chloromethane utilization *cmu* pathway (bold arrows) funnels the chloromethane-derived methyl group into central metabolism via methylene-H_4_F (CH_2_ = H_4_F), while the methanol (CH_3_OH) oxidation pathway operates with formaldehyde (HCHO) as a metabolic intermediate (grey arrows). H_4_F- and H_4_MPT-dependent enzyme-mediated steps are depicted in blue and pink, respectively. Carbon assimilation operates via the serine cycle (Ser) coupled with the ethylmalonyl-CoA pathway (EMCP) [67]. Spontaneous condensation of HCHO with H_4_F or H_4_MPT, and formaldehyde oxidation to methylene-H_4_F are shown with broken line. In the *cmu* pathway, the methyl group enters a specific H_4_F-oxidation pathway for energy production driven by the FolD and PurU enzymes. Protein-encoded genes or genes located on plasmid pCMU01 are shown in bold. Boxes and circles highlight proteins more abundant in chloromethane- and methanol grown-cultures, respectively. CmuA, methyltransferase/corrinoid-binding two-domain protein; CmuB, methylcobalamin:H_4_F methyltransferase; Fae, formaldehyde activating enzyme; Fch, methenyl-H_4_F cyclohydrolase; FDHs, formate dehydrogenases; Fhc, formyltransferase-hydrolase complex; FolD, bifunctional methylene-H_4_F dehydrogenase/cyclohydrolase; FtfL, formate-H_4_F ligase; Gck, glycerate kinase; GcvT, H_4_F-dependent aminomethyltransferase; HprA, hydroxypyruvate reductase; MDH, methanol dehydrogenase; MetF, methylene-H_4_F reductase; MtdA, bifunctional NAD(P)-dependant methylene-H_4_F and methylene-H_4_MPT dehydrogenase; MtdB, NAD(P)-dependent methylene-H_4_MPT dehydrogenase; Mch, methenyl-H_4_MPT cyclohydrolase; MtkA, malate thiokinase large subunit; MxaF, MDH alpha subunit, PurU, 10-formyl-H_4_F hydrolase; Sga, serine-glyoxylate aminotransferase [12]. Plasmid pCMU01 encoded proteins with predicted functions include putative uncharacterized methyltransferases CmuC and CmuC2, the putative PaaE-like oxidoreductase, and the putative PQQ-linked dehydrogenase of unknown specificity XoxF2. GvcT may serve to transfer methyl groups from a wide range of substrates to H_4_F, as proposed for members that belong to the COG0354-related enzymes such as YgfZ [68].

Chloromethane (CH_3_Cl) is a volatile organic compound emitted by oceans, plants, wood-rotting fungi and biomass burning, estimated to account for 17% of chlorine-catalyzed ozone degradation in the stratosphere [1]. Chloromethane-utilizing bacteria have been isolated from a wide variety of environments such as seawater, soil, sludge, and recently from plant leaf surfaces [2], and represent a potential biotic filter for chloromethane emissions. Many chloromethane degraders are facultative methylotrophic Proteobacteria [3] growing in aerobiosis with chloromethane and other C_1_ carbons such as methanol as unique source of carbon and energy. Complete and assembled genomes of two chloromethane-utilizing strains, *Methylobacterium extorquens* strain CM4 and *Hyphomicrobium* sp. strain MC1, are available [4], [5]. The only known microbial aerobic utilization pathway for chloromethane is tetrahydrofolate (H_4_F)-dependent [6]. This pathway was identified in the alpha-Proteobacterium *M. extorquens* CM4 using minitransposon random mutagenesis [7] and its chloromethane dehalogenase activity characterized in detail [8], [9]. The first step of the *cmu* (chloromethane utilization) pathway is catalyzed by the two-domain methyltransferase/corrinoid-binding CmuA protein that transfers the methyl group from chloromethane to a corrinoid cofactor [9], [10]. The methylcobalamin:H_4_F methyltransferase CmuB enzyme subsequently catalyzes the transfer of the methyl group from the corrinoid cofactor to H_4_F [8]. The H_4_F-bound C_1_ moiety of chloromethane, methylene-H_4_F (CH_2_ = H_4_F) is oxidized to carbon dioxide via formate to produce energy, or funneled into the serine pathway for biomass synthesis (Fig. 1). Evidence that H_4_F is an essential cofactor of the *cmu*-dependent degradation of chloromethane was obtained from mutant analyses in *M. extorquens* CM4, which identified *metF* (encoding methylene-H_4_F reductase) and *purU* (encoding formyl-H_4_F hydrolase) as essential genes for growth with chloromethane [10]. The pathway for chloromethane utilization in *Methylobacterium* is thus completely different from that for dichloromethane (CH_2_Cl_2_), which involves DcmA, a cytoplasmic dichloromethane dehalogenase/glutathione S-transferase yielding the central intermediate of methylotrophic metabolism formaldehyde (HCHO) [11].

Known pathways for tetrahydromethanopterin (H_4_MPT)- and H_4_F-dependent C_1_ substrate oxidation in *Methylobacterium* strains are compared in Figure 1.When growing on methanol, *M. extorquens* CM4 uses the H_4_MPT formaldehyde oxidation pathway first discovered in *M. extorquens* AM1 [12] and subsequently found to be widespread among methylotrophs.

Growth with chloromethane depends on the presence of cobalt in the medium [9] since CmuA methyltransferase activity requires a vitamin B_12_-related corrinoid cofactor that incorporates cobalt. As described for adenosylcobalamin (AdoCbl), the corrinoid cofactor may be synthesized *de novo* by one of Nature’s most complex metabolic pathways requiring around 30 enzyme-mediated steps [13], [14]. Of those, only *cobUQD* genes found adjacent to *cmu* genes have been described in *M. extorquens* CM4 [10]. Many microorganisms synthesize vitamin B_12_-related compounds from imported corrinoid intermediates [14] or from precursors such as dimethylbenzimidazole (DMB) [15] by pathways that have not been identified in chloromethane-degrading bacteria.

In this work, combined experimental and bioinformatics analysis was performed to gain a better understanding of the genes and proteins specifically associated with chloromethane utilization in *M. extorquens* CM4. A differential proteomic approach compared *M. extorquens* CM4 proteins under methylotrophic growth conditions with either chloromethane or methanol as the sole carbon and energy source. Gene clusters specific to the chloromethane response were identified, and compared to previously published clusters involved in the response of *M. extorquens* DM4 to dichloromethane [16], or involved in the methylotrophic growth of *M. extorquens* AM1 to methanol [17]. We found that growth with chloromethane elicits a specific adaptive response in *M. extorquens* CM4. In addition, the genome sequence of the chloromethane-degrading strain CM4 was compared to available complete sequences of other *M. extorquens* strains unable to grow on chloromethane (strains AM1, PA1, BJ001 and DM4; [5], [11]). Genomic analysis revealed that additional gene homologs of chromosome-encoded cognate genes for coenzyme biosynthesis, as well as specific genes such as *bluB2*, which is predicted to be involved in both H_4_F and vitamin B_12_ cofactor biosynthesis, were found nearby previously characterized genes *cmuA* and *cmuB* on a 380 kb plasmid.

## Materials and Methods

### Manual Gene Annotation and Bioinformatic Analysis

Comparative analyses were performed using the fully sequenced genomes of four representatives of the *M. extorquens* species; strain CM4 (GenBank accession no. CP001298, CP001299, CP001300), strain AM1 (GenBank accession no. CP001511, CP001512, CP001513 and CP001514), strain DM4 (GenBank accession no. FP103042 and FP103043 and FP103044), strain PA1 (GenBank accession no. CP000908) and strain BJ001 (GenBank accession no. CP001029, CP001030 and CP001031) [5]. Putative orthology relationships were operationally defined by gene pairs from different genomes satisfying an alignment threshold of at least 40% amino acid sequence identity (aa Id) over at least 80% of the length of the smallest encoded protein. The search for conserved genes clusters was performed as previously described [11]. Manual validation of automatic gene annotations on pCMU01 plasmid (also known as pMCHL01) was performed using the relational database [18] Microscope web interface (MethyloScope) https://www.genoscope.cns.fr/agc/mage/wwwpkgdb/Login/log.php?pid=26). Insertion sequence (IS) annotations were done as previously described [11]. IS elements were given names of type “ISMch3”, with “Mch” for *Methylobacterium extorquens* degrading chloromethane.

### Biological Materials, Media and Growth Conditions

The composition of Methylobacterium mineral medium M3 was adapted from that given in Vannelli et al. [7] with 0.2 g.L^−1^ (NH_4_)_2_SO_4_ final concentration and substitution of ZnCl_2_ by ZnSO_4_ in the trace element solution. M. extorquens CM4 was grown aerobically at 30°C either with chloromethane or with methanol as carbon substrate, on a rotary shaker (140 rpm) in 1.2-liter Erlenmeyer flasks containing 200 mL M3 medium, closed with gas-tight screw caps with mininert valves (Supelco). Methanol (sterile-filtered) was added to a final concentration of 40 mM. Chloromethane gas was added to a final concentration of 15 mM in the liquid phase, assuming a Henry constant of 0.0106 m^3^.atm.mol^−1^ at 30°C [19], as previously described [6]. Acetone was added to a final concentration of 5 mM from a sterile-filtered solution at 250 mM. Chloromethane and acetone degradation were quantified using a CP 3800 gas chromatograph connected to a flame ionization detector (GC-FID; Varian, USA) equipped with a GC column (CP-Sil 5 CB, length 15 m; Varian).

### Protein Extraction

Triplicates of *M*. *extorquens* CM4 cultures were harvested by centrifugation (10 min at 10,000 g) in mid-exponential growth phase of chloromethane- and methanol-grown cultures, using 100 mL at OD_600_ of 0.2 and 33 mL at OD_600_ at 0.6, respectively. Cell pellets were resuspended in 10 mM Tris, 1 mM EDTA buffer pH 7.6 (TE buffer), washed once and resuspended in 400 µL of the same buffer in the presence of benzonase (250 units; GE Healthcare) and 4 µL protease inhibitor mix 100× (GE Healthcare). Cells were disrupted using glass beads (0.1 mm in diameter, 1 g per 0.4 mL extract) in a MM2 mixer mill (Retsch Haan, Germany) at maximal speed for 6 cycles of 30 sec, and then placed on ice for one hour. Cell debris and beads were removed by centrifugation at 14,000 g for 15 min at 4°C, and the supernatant was centrifuged again at 14,000 g for one hour at 4°C. Protein concentration in the supernatant was assayed using a commercial Bradford assay (Biorad) with bovine serum albumin as a standard, and subsequently adjusted to 1 mg/mL in TE buffer.

### Two-dimensional Gel Electrophoresis (2D–E)

Protein extracts (100 µg) were precipitated overnight with 9 vol. of acetone at 4°C, centrifuged at 10,000 g for 10 min at 4°C, washed three times with 80% acetone. Proteins were resuspended in 350 µL of purified rehydration buffer (RB). RB buffer (7 M urea, 2 M thiourea) was purified by mixing for one hour with 10 g.L^−1^ Amberlite IRN-150L (GE Healthcare), 2.5% wt/vol CHAPS (3-[(3-cholamidopropyl)-dimethylammonio]-1-propanesulfonate), 0.6% immobilized pH gradient (IPG) buffer (pH range 4–7 or 3–10, respectively), 65 mM dithiothreitol (DTT), and 0.002% wt/vol bromophenol blue. Proteins (80 µg) were loaded on 18-cm IPG strips (linear gradient pH 4–7 and 3–10) with the IPGphor 3 isoelectric focusing (IEF) system, as recommended by the manufacturer (GE Healthcare). Rehydration (6 h at 0 V and 6 h at 30 V) was followed by four two-hour increments during which the voltage was increased stepwise from 150 V, 500 V, 1000 V, to 3000 V. Finally, separation was obtained using 8,000 V until a minimum of 45,000 V.h^−1^ was reached, and strips were stored at –80°C. Before use, strips were thawed at room temperature, and placed in equilibration solution (50 mM Tris-HCl, pH 8.8, 6 M urea, 30% glycerol, 2% SDS, and 0.002% bromophenol blue) twice for 15 min, first with 10 mg/mL DTT and then with 25 mg/mL iodoacetamide. The second dimension of electrophoretic separation was performed by 11.5% SDS-PAGE using the Ettan DALTII system (GE Healthcare). Gels were fixed during 1 h in a 40% ethanol, 7% acetic acid solutions. Proteins were stained overnight with Brilliant Blue G-colloidal (Sigma). Scanning of gels was performed on an Image Scanner (GE Healthcare) with LabScan software (GE Healthcare).

### Two-dimensional Fluorescence Differential Gel Electrophoresis (2D-DIGE)

Samples were labeled with CyDye DIGE Fluor minimal dyes (Cy2, Cy3 or Cy5, GE Healthcare) according to the manufacturer’s instructions. After acetone precipitation, the resulting protein pellet was resuspended in DIGE rehydration buffer (DRB) (7 M urea, 2 M thiourea, 4% wt/vol CHAPS, Tris 20 mM, pH 8.5). The protein concentration was quantified using a slightly modified Bradford method (as described above except for use of DRB), standardized with known concentrations of bovine serum albumin, and adjusted to 2 mg/mL using DRB. For each DIGE experiment, 8 samples were labeled, corresponding to four independent cultures for each condition. Labeling was performed by mixing 50 µg total protein from each of the four samples with either Cy3 (two samples) or Cy5 (two other samples) DIGE minimal dye (400 pmol) (GE Healthcare), to take into account biases resulting from different labeling efficiency. A pooled set of internal standards, comprising 25 µg aliquots from each of the 8 samples (200 µg total), was labeled with Cy2 DIGE minimal dye (1600 pmol total). Labeling was performed for 30 minutes on ice and in the dark, and quenched by the addition of 10 mM lysine (1 µL for Cy3- or Cy5-labeled extracts, and 4 µL for Cy2-labeled extracts, respectively). Samples were incubated for 10 min on ice in the dark. Finally, protein samples that were separated on the same gel were mixed (one Cy3- and one Cy5-labeled sample each together with one-fourth volume of the pooled set of internal standards), and supplemented with 0.6% IPG pH 3–10 NL (non-linear) and 65 mM DTT. Buffer RB was added to each mix to final volume of 350 µL before IEF separation. Proteins were loaded on 18-cm IPG strips (non-linear gradient pH 3–10) and submitted to separation steps as described above. Gels were fixed and proteins stained as described above. Scanning of gels was performed with an Ettan DIGE Imager (GE Healthcare).

### Proteome Image Analysis

Differential analysis was performed using ImageMaster 2D Platinum software (v. 6.0, GE Healthcare). Six gels were grouped in two classes of three independent gels depending on the two compared conditions (chloromethane or methanol growth conditions). Gels were matched with one reference gel (master gel) following spot detection. For each spot, the relative volume corresponded to the normalized volume of the spot compared to the normalized volume of the entire gel coloration. Statistical analysis was performed by calculating the Student *t* value for each spot, as well as a ratio value defined as the mean of the relative volume of the spot obtained in the different replicates for growth with chloromethane divided by the mean of the relative volumes obtained for growth with methanol. Spots with a Student *t* value higher than 1.9 (corresponding to a *p*-value of <0.1) and ratios ≥2.0 or ≤−2.0 were analyzed by mass spectrometry. DIGE images were analyzed with DeCyder software (v. 7.0, GE Healthcare). A total of twelve images obtained from 4 gels (three images each) were analyzed. Student’s *t* test was used to determine differential abundance of proteins. In this procedure, the *p*-values were corrected for false discovery rate [20]. Spots with a *p*-value <0.01 and ratios ≥2.0 or ≤−2.0 were considered to be differentially abundant.

### Mass Spectrometry Protein Identification

The procedure of Muller *et al*. [16] was followed for spot identification, with minor adjustments. Mass spectrometry analyses were performed in reflector positive mode on a Biflex III (Bruker-Daltonik GmbH, Bremen, Germany) matrix-assisted laser desorption/ionization time-of-flight mass spectrometer (MALDI-TOF TOF) and on an Autoflex III Smartbeam (Bruker-Daltonik GmbH, Bremen, Germany) matrix-assisted laser desorption/ionization time-of-flight mass spectrometer (MALDI-TOF TOF). A saturated solution of α-cyano-4-hydroxycinnamic acid in 50% water/50% acetonitrile was used as matrix for MALDI mass measurement on the Biflex III. Peptide mass fingerprinting data (PMF) and peptide fragment fingerprinting data (PFF) were combined by Biotools 3 software (Bruker Daltonik) and transferred to the search engine MASCOT (Matrix Science, London, UK). Peptide mass error was limited to 100 ppm for the Biflex III and to 50 ppm for the Autoflex III Smartbeam. Proteins were identified by searching against the NCBI non-redundant protein sequence database and the predicted proteins of strain CM4 (GenBank accession no. CP001298, CP001299, CP001300).

## Results

### The 380 kb Episome in *M. extorquens* CM4 Harbors *cmu* Genes and Associated Genes

The repertoire of known *cmu* genes and genes conserved in chloromethane-degrading strains includes genes essential for dehalogenation of chloromethane (*cmuA*, *cmuB*), genes essential for growth with chloromethane as the sole carbon and energy source (*cmuC*, *metF*, *purU*), and genes found in the vicinity of genes *cmuA, cmuB and cmuC* in methylotrophic chloromethane-degrading strains (*fmdB*, *paaE*, *hutI*, and *cbiD*) [2]. All these genes co-localize on a 138 kb region flanked by transposable elements nested within the 380 kb plasmid pCMU01 in *M. extorquens* CM4. This plasmid encodes proteins associated with growth on chloromethane, such as the enzymes for chloromethane degradation and for metabolism of the two essential dehalogenase cofactors AdoCbl and H_4_F, as well as transport proteins for coenzyme B_12_ precursors (Table 1). Thus, plasmid pCMU01 can be designated as a chloromethane catabolic plasmid that harbors the cognate essential genes for growth on chloromethane.

**Table 1:** Analysis of the theoretical proteome of plasmid pCMU01.

Functional class	Occurrence in sequenced *Methylobacterium extorquens* genomes^a^		
	Unique	Common	Occasional
Chloromethane degradation	6(CmuA, CmuB, CmuC, CmuC2^b^, HutI, PaaE-like)	0	0
Cobalamin metabolism	3(BluB2, CbiD, CobA)	13(Cob protein)	0
H_4_F and C_1_ metabolism	3(FolD, MetF2 c, PurU)	4(FolC2, Hss2, SerC2, XoxF2)	0
Acetone degradation	3(AcxA, AcxB, AcxC)	0	0
Other metabolisms	7	10 (Gck2, Shc2)	19 (GdhA, IspF, SorA, SorB)
Stress	0	1 (UspA fragment)	1 (Usp) d
Plasmid-related function	3 (MmeI)	1 (ArdC)	19 (DotABC-like, IcmBCEKL, TraGDCA, RepABC)
Transporter	3	10 (BtuC, BtuF, BtuD, ClcA, ModA2, ModB2, ModC2, Mop2)	7 (BtuB, CzcBA2 e)
Regulator	14 (AcxR, FmdB)	8	12 (CzcSR e)
Mobile element-related	25	3	43
Unknown	90	6	72

a Compared predicted proteome sizes are, *M. extorquens* strains AM1, 6531 proteins (genome sequence accession no NC_012808); DM4, 5773 proteins (NC_012988); PA1, 5357 proteins (NC_01017); CM4, 6454 proteins (NC_011757); BJ001, 6027 proteins (NC_010725). Homologous proteins were defined as proteins with at least 40% identity covering over 80% of the sequence. Three classes of proteins were considered: Unique, 157 pCMU01 plasmid-encoded proteins without homologs in any of the compared genomes, including the chromosome and the second plasmid p2MCHL of strain CM4; Common, 56 pCMU01 plasmid-encoded proteins with homologs on the chromosome of all 5 *M. extorquens* genomes including that of strain CM4; Occasional, 173 pCMU01 plasmid-encoded proteins with homologs in at least one of the 5 *M. extorquens* genomes. Plasmid pCMU01 and plasmid p1METDI of strain DM4 share 56 homologs localized on three gene clusters. Selected examples are indicated when relevant.

b CmuC/CmuC2 homologs share less homologies between them (31% aa Id) than with homologs found in other chloromethane-degrading *Hyphomicrobium* strains: 40% with strain CM2 CmuC [71] and 37% aa Id with strain MC1 CmuC [4]. *M. extorquens* CM4 is the only chloromethane-degrading strain so far which contains two methyltransferase-encoding *cmuC* genes of unknown function. Transposon insertion in gene *cmuC* was previously demonstrated to prevent strain CM4 growth with chloromethane [10].

c pCMU01 plasmid encoded protein MetF2 (Mchl_5726) previously demonstrated to be essential for chloromethane utilization [6] encodes a protein with only 25% aa Id to *E*. *coli* MetF. It is more distantly related to the canonical MetF than its chromosomal homolog (Mchl_1881, 56% aa Id to *E. coli* MetF).

d Putative universal stress protein (Mchl_5472) also found in the DCM-dehalogenating *M. extorquens* DM4 only (METDI4473).

e Close homologs (>65% Id aa) located in synteny on the 1.26 Mb megaplasmid of strain AM1.

### Overview of Plasmid pCMU01 Gene Content

Plasmid pCMU01 is characterized by a somewhat lower GC content (66.3%) than the chromosome of strain CM4 (68.2%). To a large extent (41%), it features unique genes encoding predicted proteins without close homologs (>40% aa Id, >80% of the protein length) in the genomes of four other *M. extorquens* strains unable to degrade chloromethane [5] (Table 1). Of its 386 predicted CDS, 56% belong to at least one COG group [21] related to metabolism (enzymes, 69 CDS; transporters, 20 CDS), plasmid functions (23 CDS), adaptive response (regulation, 34 CDS; stress, two CDS), and genomic plasticity (mobile DNA elements, 71 CDS with a total of 18 identified IS elements representing 4% of the predicted CDS of the plasmid). Pseudogenes may account for 9% of the total predicted CDS on the plasmid with 35 pseudogenes detected.

Chloromethane is not the only organic molecule for which the plasmid allows to transform for growth. A complete acetone-catabolic gene cluster encoding the acetone carboxylase subunits (β subunit, *acxA*; α subunit, *acxB*; γ subunit, *acxC*) and its cognate transcriptional activator (gene *acxR*) was found. Acetone carboxylase is the key enzyme of bacterial acetone metabolism in *Xanthobacter autotrophicus* strain Py2, catalyzing the ATP-dependent carboxylation of acetone to form acetoacetate [22]. High sequence conservation was found between the *acxRABC* gene clusters of strains CM4 and *X. autotrophicus* Py2 (>82% aa Id for enzyme subunits and 53% for the regulator). The ability of *M. extorquens* CM4 to degrade acetone was tested in aerobic liquid cultures in M3 medium. When 5 mM acetone was supplied as the unique source of carbon and energy, strain CM4 grew up to an OD_600_ of 0.4 at stationary growth phase, with total degradation of acetone as measured using GC-FID. No acetone degradation was observed in the abiotic control or in cultures of *M. extorquens* DM4 lacking the *acx* cluster under the same conditions. Thus, the plasmid pCMU01-encoded *acx* cluster seems to be functional in strain CM4.

Evidence of the mode of replication, maintenance, and conjugation of plasmid pCMU01 was suggested from sequence similarity searches. A combined replication and partitioning *repABC* unit (Mchl_5615–5617) including the incompatibility antisense RNA (ctRNA) between the *repB*-*repC* genes was found [23]. The gene products of *repA*, *repB* and *repC* share at least 43% aa Id with the corresponding proteins of the characterized *Rhizobium etli* p42d plasmid *repABC* cassette [24]. In a recent review, plasmid pCMU01 was classified within the RepABC family plasmids of large low-copy-number plasmids found exclusively in Alphaproteobacteria [25]. The plasmid harbors components of a core type IVB secretion/conjugation system complex often used for horizontal propagation, including the gene encoding the conserved central component of the DNA transport activity core complex (Mchl_5595), and *traD* (Mchl_5572) which lies within a *traGDCA* gene cluster (Mchl_5572–5575) conserved in other Alphaproteobacteria plasmids including *Agrobacterium tumefaciens* Ti plasmids (encoded proteins TraG, D, C and A sharing 43, 53, 38 and 44% aa Id, respectively). Finally, a putative restriction- modification system encoding protein Mchl_5634 shares 48% aa Id with a bifunctional DNA methyltransferase/type II restriction endonuclease MmeI [26] (Table 1). Taken together, the described genomic features indicate that plasmid pCMU01 represents a low-copy plasmid, vertically transmitted via a RepABC replication and partitioning unit, and most probably able to propagate by horizontal transfer within Alphaproteobacteria.

### Extensive Plasmid-encoded Gene Redundancy Associated with Vitamin B_12_ Metabolism

*M. extorquens* CM4 is able to synthesize coenzyme B_12_, a cofactor essential for activity of chloromethane dehalogenase CmuAB [9]. A complete set of *cob* genes homologous to those described in *P. denitrificans* for the aerobic biosynthesis pathway of AdoCbl [14] is found on the chromosome of CM4 as well as on the chromosomes of four other *M. extorquens* strains (Table 2). Remarkably, strain CM4 also contains plasmid-borne copies of 13 *cob* genes and genes coding for cobalt and preformed corrinoid transporters beyond to the close chromosomal homologs of these genes shared by *M. extorquens* strains. These include the putative cobalt transporter CzcA-related RND transporter [27] (the plasmid-borne gene product Mchl_5715 displays 43% aa Id with the chromosome-encoded Mchl_1072; Table 2), and a homolog of the preformed corrinoid specific transporter Btu [28]. Unlike the plasmid-borne *btu* gene cluster, the chromosome-encoded *btuFCD* cluster lacks the *btuB* gene preceded by a cobalamin riboswitch [29] (Mchl_misc_RNA_1, Table 2), suggesting that expression of the plasmid-borne *btu* gene cluster is controlled by cobalamin in its coenzyme form (AdoCbl).

**Table 2:** Gene redundancy for cobalamin and tetrahydrofolate metabolism in *M. extorquens* CM4.

Function	Gene in strain CM4				Occurrence in *M. extorquens* a	MaGe annotation^b^		EC n°		Plasmid pCMU01 identifier			
	Chromosome	Plasmid pCMU01	Paralog aa Id (%)										
***Cobalamin metabolism***													
**Aerobic AdoCbl biosynthesis from precursors** c													
	*bluB*	*bluB2*	38.4		core	5,6-dimethylbenzimidazole synthase (flavin destructase), putative cob(II)yrinic acid a,c-diamide reductase			1.16.8.1		Mchl_5732		
	/d	*cbiD*	/		CM4 specific	Cobalamin biosynthesis protein, putative cobalt-precorrin-6A synthase [deacetylating]			2.1.1.-		Mchl_5729		
	/	*cobA* e	/		CM4 specific	S-adenosyl-L-methionine-dependent uroporphyrinogen III methylase (SUMT)			2.1.1.107		Mchl_5731		
	*cobB*	/	/		core	Cobyrinic acid a,c-diamide synthase					/		
	*cobC*	*cobC2* e	51.2		core	L-threonine-O-3-phosphate decarboxylase			4.1.1.81		Mchl_5730		
	*cobD*	*cobD2*	76.4		core	Cobalamin biosynthesis protein CobD					Mchl_5724		
	*cobE*	*cobE2*	64.4		core	Cobalamin biosynthesis protein CobE					Mchl_5686		
	*cobF*	/	/		core	Precorrin-6A synthase			2.1.1.152		/		
	*cobG*	/	/		core	Putative precorrin-3B synthase CobG			1.14.13.83		/		
	*cobH*	*cobH2*	86.2		core	Precorrin-8X methylmutase			5.4.1.2		Mchl_5691		
	*cobI*	*cobI2*	80.7		core	Precorrin-2 C(20)-methyltransferase			2.1.1.130		Mchl_5690		
	*cobJ*	*cobJ2*	79.1		core	Precorrin-3B C(17)-methyltransferase			2.1.1.131		Mchl_5689		
	*cobK*	*cobK2*	63.4		core	Precorrin-6A reductase			1.3.1.54		Mchl_5688		
	*cobL*	*cobL2*	76.0		core	Precorrin-6Y C(5,15)-methyltransferase			2.1.1.132		Mchl_5687		
	*cobM*	*cobM2*	82.7		core	Precorrin-4 C(11)-methyltransferase			2.1.1.133		Mchl_5685		
	*cobN*	/	/		core	Putative cobaltochelatase, CobN-related			6.6.1.2		/		
	*cobO*	*cobO2*	83.5		core	Cob(I)yrinic acid a,c-diamide adenosyltransferase			2.5.1.17		Mchl_5722		
	*cobP*	*cobP2*	73.6		core	Bifunctional adenosylcobalamin biosynthesis protein CobP			2.7.7.62		Mchl_5721		
	*cobQ*	*cobQ2*	75.2		core	Cobyric acid synthase					Mchl_5723		
	*cobS*	/	/		core	Aerobic cobaltochelatase subunit CobS			6.6.1.2		/		
	*cobT*	/	/		core	Aerobic cobaltochelatase subunit CobT			6.6.1.2		/		
	*cobU*	*cobU2*	54.5		core	Nicotinate-nucleotide–dimethylbenzimidazole phosphoribosyltransferase			2.4.2.21		Mchl_5702		
	*cobV*	/	/		core	Cobalamin synthase			2.-.-.-		/		
	*cobW*	/	/		core	Cobalamin biosynthesis protein CobW					/		
	*cobW*	/	/		core	Putative cobalamin biosynthesis protein CobW					/		
	*cobW*	/	/		core	Putative cobalamin biosynthesis protein CobW					/		
	*cysG*	/e	/		core	Siroheme synthase			2.1.1.107/1.3.1.76/4.99.1.4		/		
**Cobalt and cobalamin transporters**													
	/	*btuB*	/		accessory	Putative vitamin B_12_ outer membrane transporter BtuB					Mchl_5676		
	*btuC*	*btuC2*	73.5		core	Putative vitamin B_12_ import system permease protein BtuC					Mchl_5678		
	*btuD*	*btuD2*	66.0		core	Putative vitamin B_12_ transport system BtuD, ATPase component					Mchl_5679		
	*btuF*	*btuF2*	61.0		core	Putative vitamin B_12_-binding protein BtuF			1.16.8.1		Mchl_5677		
	*cbtA*	/	/		core	Putative cobalt transporter, subunit CbtA					/		
	*cbtB*	/	/		core	Putative cobalt transporter, subunit CbtB					/		
	*corA*	/	/		core	Putative cobalt transporter CorA					/		
	*czcA*	*czcA2*	43.4		core	RND efflux transporter, membrane component, cobalt-zinc-cadmium resistance protein					Mchl_5715		
	/	*czcB*	/		accessory	RND efflux transporter, membrane fusion protein, putative CzcB protein					Mchl_5714		
	*exbB*	/	/		core	Transport protein ExbB					/		
	*exbD*	/	/		core	Transport protein ExbD					/		
	*icuA*	/	/		core	TonB-dependent outer membrane transporter associated to improved cobalt uptake					/		
	*icuB*	/	/		core	Periplasmic binding protein associated to improved cobalt uptake					/		
	*icuC*	/	/		core	Putative periplasmic binding protein; improves cobalt uptake when overexpressed					/		
	*tolQ*	/	/		core	Transport protein ExbB/TolQ					/		
	*tolR*	/	/		core	Transport protein ExbD/TolR					/		
	*tonB*	/	/		core	Putative TonB family protein					/		
***Tetrahydrofolate metabolism***													
*** de novo*** ** tetrahydrofolate biosynthesis** f													
	*dmrA*	/	/		core	Dihydromethanopterin reductase, putative dihydrofolate reductase					/		
	*folA*	/	/		core	Dihydrofolate reductase (also called *dfrA*)			1.5.1.3		/		
	*folB*	/	/		core	Dihydroneopterin aldolase			4.1.2.25		/		
	*folC*	*folC2*	46.3		core	Bifunctional folylpolyglutamate synthase/dihydrofolate synthase			6.3.2.17/6.3.2.12		Mchl_5701		
	*folE*	/	/		core	GTP cyclohydrolase I			3.5.4.16		/		
	*folK*	/	/		core	2-amino-4-hydroxy-6-hydroxymethyldihydropteridin pyrophosphokinase			2.7.6.3		/		
	*folP*	/	/		core	Dihydropteroate synthase			2.5.1.15		/		
	Mchl_0356	/	/		core	NUDIX hydrolase (NudG), putative dihydroneopterin triphosphate pyrophosphatase (NtpA-like)					/		
	*pabA*	/	/		core	Aminodeoxychorismate synthase subunit II, p-aminobenzoate synthase component			2.6.1.85		/		
	*pabB*	/	/		core	Para-aminobenzoate synthase component I			2.6.1.85		/		
	*pabC*	/	/		core	Putative 4-amino-4-deoxychorismate lyase component of para-aminobenzoate synthase			4.1.3.38		/		
** D-erythrose-4P to chorismate**													
	*aroA*	/	/		core	3-enolpyruvylshikimate-5-phosphate synthetase			2.5.1.19		/		
	*aroC*	/	/		core	Chorismate synthase			4.2.3.5		/		
	*aroE*	/	/		core	Putative shikimate 5-dehydrogenase			1.1.1.25		/		
	*aroG*	/	/		core	2-dehydro-3-deoxyphosphoheptonate aldolase			4.1.2.54		/		
	*aroK*	/	/		core	Putative transcriptional regulator (N-terminal)/shikimate kinase (C-terminal)			2.7.1.71		/		
	*aroQ*	/	/		core	3-dehydroquinate dehydratase, type II			4.2.1.10		/		
	Mchl_1923	/	/		core	Bifunctional shikimate kinase (AroK)/dehydroquinate synthase (AroB)			4.2.3.4		/		
** D-erythrose-4P synthesis from sugars**													
	*cbbA*	/	/		core	Fructose-bisphosphate aldolase			4.1.2.13		/		
	*fbp*	/	/		core	Fructose-1,6-bisphosphatase I			3.1.3.11		/		
	*glpX*	/	/		core	Fructose 1,6-bisphosphatase II			3.1.3.11		/		
	*tpiA*	/	/		core	Triosephosphate isomerase			5.3.1.1		/		
** Tetrahydrofolate interconversion**													
	/	*folD*	/	CM4 specific			Bifunctional methylene-H_4_F dehydrogenase/methenyl-H_4_F cyclohydrolase		1.5.1.5/3.5.4.9		Mchl_5700		
	*ftfL*	/	/	core			Formate-H_4_F ligase		6.3.4.3		/		
	*gcvH*	/	/	core			Glycine cleavage complex protein H				/		
	*lpd* f	/	/	core			Glycine-cleavage complex protein L (dihydrolipoamide dehydrogenase)		1.8.1.4		/		
	*gcvP*	/	/	core			Glycine cleavage complex protein P, PLP-dependent glycine dehydrogenase		1.4.4.2		/		
	*gcvT*	/	/	core			Glycine cleavage complex protein T, H_4_F-dependent aminomethyltransferase		2.1.2.10		/		
	*glyA*	/	/	core			Serine hydroxymethyltransferase		2.1.2.1		/		
	*metF*	*metF2*	26	core			5,10-methylene-H_4_F reductase		1.5.1.20		Mchl_5726		
	*purH*	/	/	core			Bifunctional IMP cyclohydrolase/phosphoribosyl-aminoimidazolecarboxamide formyltransferase		3.5.4.10/2.1.2.3		/		
	*purN*	*purU* e	32	core			Phosphoribosylglycinamide formyltransferase 1		2.1.2.2		Mchl_5699		

a Homologs with >90% aa Id (with mentioned exceptions) found in the chromosome of all *M. extorquens* strains AM1, BJ001, DM4, and PA1 (common core genome), in one of the strains (shared accessory genome), or none of these strains (CM4 specific CDS). The accessory genome includes a *btuB* homolog (Mpop_3807, 65% aa Id) in strain BJ001. For strain AM1, a putative dihydrofolate reductase *dfrB* gene (META2_0242, 34 and 28% aa Id with DmrA and DfrA, respectively) is found in addition to the chromosomal gene; moreover, homologs to Mchl_1923 (META2_0462, 33% aa Id with the N-terminal domain), and CzcA2 (META2_1026, 85% aa Id with pCMU01 plasmid *czcA2*) are found.

b MaGe annotation (https://www.genoscope.cns.fr/agc/microscope).

c Precursors are uroporphyrinogen III and 5,6-dimethylbenzimidazole.

d n.d., not detected.

e Encode for homologs of different length: CobA (267 aa)/CysG (485 aa); CobC2 (519 aa)/CobC (338 aa); PurU (287 aa)/PurN (219 aa).

f In *M. extorquens* strains, H_4_F is synthesized either *de novo* or salvaged from 5,10-methenyl-H_4_F, or 5- or 10-formyl-H_4_F [11], [72], [73].

### Experimental Identification of Gene Clusters Specific of the Chloromethane Response

Differential analyses of proteins extracted from chloromethane- and methanol-grown cultures of *M. extorquens* CM4 were performed using 2D-E and 2D-DIGE. Overall, 88 protein spots showing differences in abundance between the two compared conditions were detected, resulting in the identification of 49 proteins (Table 3; Fig. S1). In total, 33 proteins were specific of chloromethane-grown cultures, whereas sixteen proteins were more abundant in methanol-grown cultures.

**Table 3:** Proteomic analysis of differentially expressed proteins in chloromethane- and methanol-grown cultures of *M. extorquens* CM4.

Protein	Identifier^a^	Gene	Protein parameters			Mass spectrometry identification data b of different p*I* ranges tested								
			Ratio c CH_3_Cl/CH_3_OH	*M*_r_ (kDa)	p*I*	4–7			3–10			3–10 NL d		
						Score	Error (ppm)	Coverage (%)	Score	Error (ppm)	Coverage (%)	Score	Error (ppm)	Coverage (%)
***Chloromethane utilization***														
CmuA, two-domain methyltransferase/corrinoid binding protein	Mchl_5697	*cmuA* e	CH_3_Cl f	67.0	5.5	223	54	33	203	37	50	224	47	48
CmuB, methylcobalamin:H_4_F methyltransferase (EC 2.1.1.86)	Mchl_5727	*cmuB* e	CH_3_Cl f	33.3	5.1	203	21	57	142	27	47	130	33	49
CobH2, precorrin-8X methylmutase (EC 5.4.1.2)	Mchl_5691^g^	*cobH_2_* e	CH_3_Cl	22.0	5.1	203	28	85	n.d. h	n.d.	n.d.	n.d.	n.d.	n.d.
MetF, 5,10-methylene-H_4_F reductase (EC 1.5.1.20)	Mchl_1881	*metF*	CH_3_Cl	34.1	6.6	n.d.	n.d.	n.d.	170	63	47	n.d.	n.d.	n.d.
PaaE-like, oxidoreductase FAD/NAD(P)-binding domain protein	Mchl_5717	*paaE* e	CH_3_Cl/+++ f	40.2	4.7	258	27	66	271	51	64	216	10	65
PurU, formyl-H_4_F hydrolase (EC 3.5.1.10)	Mchl_5699	*purU* e	CH_3_Cl f	32.8	6.6	n.d.	n.d.	n.d.	238	40	66	147	13	61
***Methylotrophy***														
Fae, formaldehyde-activating enzyme (EC 4.3.−. − )	Mchl_2169	*fae*	− −	18.1	5.7	108	47	42	n.d.	n.d.	n.d.	n.d.	n.d.	n.d.
Fch, methenyl-H_4_F cyclohydrolase (EC 3.5.4.9)	Mchl_2134	*fch*	− − f	21.7	4.8	n.d.	n.d.	n.d.	n.d.	n.d.	n.d.	116	18	55
FtfL, formate-H_4_Fligase (EC 6.3.4.3)	Mchl_0447	*ftfL*	+++	59.5	6.8	n.d.	n.d.	n.d.	348	46	58	n.d.	n.d.	n.d.
Hpr, hydroxypyruvate reductase, NAD(P)H-dependent (EC 1.1.1.29)	Mchl_2132	*hprA*	− −	34.2	5.2	152	46	39	n.d.	n.d.	n.d.	n.d.	n.d.	n.d.
MauB, methylamine dehydrogenase (EC 1.4.99.3) large subunit	Mchl_0565	*mauB*	CH_3_Cl/+++	44.7	7.2	n.d.	n.d.	n.d.	270	49	66	168	11	49
MtdA, bifunctional protein [NADP-dependent methylene-H_4_MPT/methylene-H_4_Fdehydrogenase] (EC 1.5.1.−/1.5.1.5)	Mchl_2133	*mtdA*	− −	29.7	7.0	n.d.	n.d.	n.d.	n.d.	n.d.	n.d.	121	43	34
MtkA, malate thiokinase large subunit (EC 6.2.1.9)	Mchl_2135	*mtkA*	**−** −	42.0	5.8	n.d.	n.d.	n.d.	278	51	75	n.d.	n.d.	n.d.
MxaF, methanol dehydrogenase (EC 1.1.99.8) large subunit	Mchl_4518	*mxaF*	CH_3_Cl/+++ f	68.4	5.9	129	16	19	364	63	54	82	8	20
Sga, serine glyoxylate aminotransferase (EC 2.6.1.45)	Mchl_2131	*sga*	**−** **−** f	43.2	6.9	n.d.	n.d.	n.d.	n.d.	n.d.	n.d.	283	24	78
***Central metabolism***														
Acs, acetyl-CoA synthetase (EC 6.2.1.1)	Mchl_2785	*acs*	CH_3_Cl/+++ f	72.2	5.6	413	38	53	374	53	55	231	16	41
CbbA, fructose-bisphosphate aldolase (EC 4.1.2.13)	Mchl_2646	*cbbA*	CH_3_Cl	38.6	5.5	92	37	28	n.d.	n.d.	n.d.	n.d.	n.d.	n.d.
CysK, cysteine synthase A and O-acetylserine sulfhydrolase A subunit (EC 2.5.1.47)	Mchl_0937	*cysK*	CH_3_Cl	34.5	5.9	186	37	64	n.d.	n.d.	n.d.	n.d.	n.d.	n.d.
EtfA, electron transfer flavoprotein subunit alpha	Mchl_1823	*etfA*	**−** **−**	32.4	5.0	n.d.	n.d.	n.d.	190	57	76	n.d.	n.d.	n.d.
EtfB, electron transfer flavoprotein subunit beta	Mchl_1822	*etfB*	**−** **−**	26.7	7.9	n.d.	n.d.	n.d.	n.d.	n.d.	n.d.	90	7	37
FumC, fumarase C (EC 4.2.1.2)	Mchl_2891	*fumC*	CH_3_Cl	49.8	5.6	129	81	51	n.d.	n.d.	n.d.	n.d.	n.d.	n.d.
GcvT, H_4_F-dependent aminomethyltransferase, glycine cleavage complex subunit (T protein) (EC 2.1.2.10)	Mchl_0814	*gcvT*	CH_3_Cl	40.3	6.0	n.d.	n.d.	n.d.	189	42	55	n.d.	n.d.	n.d.
GlpX, fructose 1,6-bisphosphatase, class II (EC 3.1.3.11)	Mchl_2242	*glpX*	+++	34.6	5.4	146	44	41	n.d.	n.d.	n.d.	n.d.	n.d.	n.d.
HisA, phosphoribosylformimino-5-aminoimidazole carboxamide ribotide isomerase	Mchl_2774	*hisA*	CH_3_Cl	26.7	5.4	183	27	42	n.d.	n.d.	n.d.	n.d.	n.d.	n.d.
HisD, bifunctional histidinal dehydrogenase and histidinol dehydrogenase (EC 1.1.1.23)	Mchl_2261	*hisD*	CH_3_Cl	45.4	5.0	n.d.	n.d.	n.d.	200	34	58	n.d.	n.d.	n.d.
Hss, homospermidine synthase (EC 2.5.1.44)	Mchl_5462 i	*hss2* e	**−** **−**	53.2	5.3	246	37	40	n.d.	n.d.	n.d.	103	9	25
Lpd, dihydrolipoamide dehydrogenase (EC 1.8.1.4), glycine cleavage complex	Mchl_1930	*lpd*	**−** **−** **−**	49.0	5.7	81	26	20	n.d.	n.d.	n.d.	n.d.	n.d.	n.d.
MetK, S-adenosylmethionine synthetase (EC 2.5.1.6)	Mchl_3629	*metK*	**−** **−**	41.8	5.4	n.d.	n.d.	n.d.	n.d.	n.d.	n.d.	227	19	76
NAD(P)H:quinone oxidoreductase (EC 1.6.5.2)	Mchl_4391	*qorB*	CH_3_Cl	38.2	7.8	254	31	63	n.d.	n.d.	n.d.	n.d.	n.d.	n.d.
NuoE, NADH-quinone oxidoreductase, chain E (EC 1.6.5.3)	Mchl_1210	*nuoE*	++	44.6	4.8	n.d.	n.d.	n.d.	139	57	65	n.d.	n.d.	n.d.
NuoF, NADH-quinone oxidoreductase, chain F (EC 1.6.5.3)	Mchl_1209	*nuoF*	CH_3_Cl	47.4	6.4	n.d.	n.d.	n.d.	217	30	49	n.d.	n.d.	n.d.
PntAA, NAD(P)+ transhydrogenase, subunit alpha part 1 (EC 1.6.1.2)	Mchl_2986	*pntAA*	CH_3_Cl/+++ f	39.6	5.6	177	35	45	n.d.	n.d.	n.d.	256	18	83
ureidoglycolate lyase (EC 4.3.2.3)	Mchl_4377	j	CH_3_Cl	31.4	5.3	70 l	24 l	25 l	n.d.	n.d.	n.d.	n.d.	n.d.	n.d.
putative homoserine O-acetyltransferase (EC 2.3.1.31)	Mchl_4434	j	++	42.1	5.9	n.d.	n.d.	n.d.	279	27	66	n.d.	n.d.	n.d.
***Adaptation to stress***														
KatA, catalase (hydroperoxidase II) (EC 1.11.1.6)	Mchl_3534	*katA*	CH_3_Cl/+++ f	60.0	5.9	394	23	63	324	35	57	256	6	56
MdoG, periplasmic glucan biosynthesis protein	Mchl_2321	*mdoG*	CH_3_Cl	58.7	5.6	n.d.	n.d.	n.d.	323	47	56	n.d.	n.d.	n.d.
RfbC, dTDP-4-dehydrorhamnose 3,5-epimerase (EC 5.1.3.13)	k	*rfbC*	+++	19.8	5.5	102	25	50	n.d.	n.d.	n.d.	n.d.	n.d.	n.d.
SufS, selenocysteine lyase (EC 4.4.1.16)	Mchl_4348	*sufS*	+++	45.6	5.9	223	33	65	n.d.	n.d.	n.d.	n.d.	n.d.	n.d.
SurE, 5′-nucleotidase (EC 3.1.3.5)	Mchl_4603	*surE*	CH_3_Cl	27.3	5.4	112	27	46	n.d.	n.d.	n.d.	n.d.	n.d.	n.d.
UspA-like, putative universal stress protein	Mchl_1555	*uspA*	+++	29.3	6.1	n.d.	n.d.	n.d.	n.d.	n.d.	n.d.	79	10	32
putative manganese catalase (EC 1.11.1.6)	Mchl_3002	j	CH_3_Cl	31.0	4.9	144	36	42	n.d.	n.d.	n.d.	n.d.	n.d.	n.d.
***Protein biosynthesis and modification***														
AspS, aspartyl-tRNA synthetase (EC 6.1.1.12)	Mchl_4374	*aspS*	CH_3_Cl	67.2	5.5	n.d.	n.d.	n.d.	245	16	48	n.d.	n.d.	n.d.
ClpP, ATP-dependent Clp protease, proteolytic subunit (EC 3.4.21.92)	Mchl_2679	*clpP*	**−** **−**	23.1	5.8	n.d.	n.d.	n.d.	181	40	71	n.d.	n.d.	n.d.
EF-Ts, protein chain elongation factor	Mchl_2348	*tsf*	**−** **−**	32.3	5.5	n.d.	n.d.	n.d.	214	27	70	n.d.	n.d.	n.d.
EF-Tu, protein chain elongation factor, GTP-binding factor	Mchl_2438	*tufB*	**−** **−** f	43.1	5.4	n.d.	n.d.	n.d.	86	32	28	n.d.	n.d.	n.d.
***Other functional class***es														
ABC transporter, sulfate/thiosulfate transporter periplasmic protein	Mchl_0592	*cysP*	CH_3_Cl	30.9	5.1	n.d.	n.d.	n.d.	254	31	63	n.d.	n.d.	n.d.
ABC transporter, putative periplasmic substrate-binding protein	Mchl_0388	j	CH_3_Cl	69.0	6.7	288	45	52	n.d.	n.d.	n.d.	n.d.	n.d.	n.d.
ABC transporter, putative substrate-binding protein, aliphatic sulphonates	Mchl_0381	j	**−** **−**	34.3	8.1	n.d.	n.d.	n.d.	n.d.	n.d.	n.d.	120	8	47
conserved protein of unknown function	Mchl_4437	j	**−** **−**	16.1	5.5	n.d.	n.d.	n.d.	n.d.	n.d.	n.d.	234 l	21 l	26 l

a MaGe database (http://www.genoscope.cns.fr/agc/mage).

b Probability-based mowse score calculated using MASCOT software (Matrix Science, London, UK); error refers to mass accuracy; coverage refers to the percentage of the protein sequence covered by the matched peptides.

c Spots indicated as “CH_3_Cl” were only detected in the proteome of *M. extorquens* CM4 grown with chloromethane. Spots indicated as “+” were more abundant in chloromethane-grown cultures (or less abundant in methanol-grown cultures). Spots indicated as “−” were more abundant in methanol-grown cultures (i.e. less abundant in chloromethane-grown cultures). Factors of differential abundance were defined as follows:++(**−−**) 2- to 5-fold;+++(**−−**−) more than 5-fold.

d NL, non linear p*I* range used in 2D-DIGE experiments.

e Only found in strain CM4 (among the 8 *Methylobacterium* strains for which the complete genome sequence is known; [5], [11]) and localized on plasmid pCMU01.

f Multiple spots detected.

g Mass spectrometry used to discriminate from Mchl_1712 displaying 86% sequence identity at the protein level.

h n.d., not detected.

i Mass spectrometry used to discriminate from Mchl_2317 displaying 96% sequence identity at the protein level.

j No assigned gene name.

k Mass spectrometry data did not allow us to discriminate between two homologs with 99% sequence identity (Mchl_2669/Mchl_4004).

l Tandem mass spectrometry identification.

**Figure 2:**
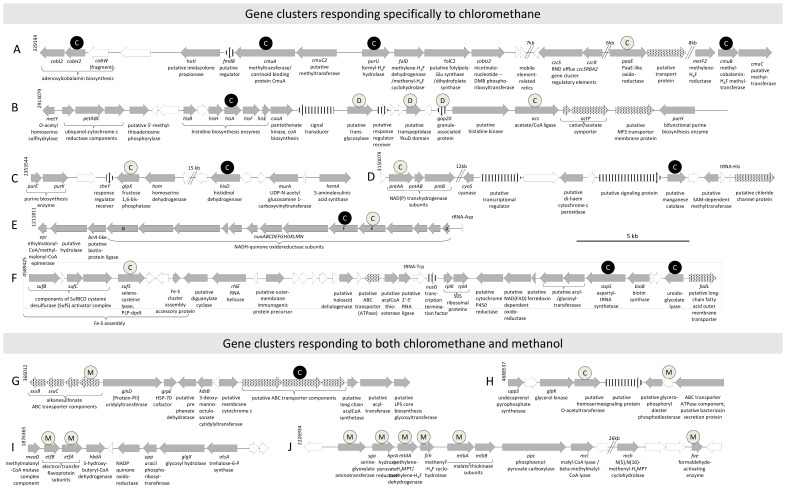
Gene clusters associated with the chloromethane response. Sequence positions are indicated for each gene cluster. All but cluster A are located on the chromosome. Some DNA segments are omitted for clarity (double slashes), with their size indicated in kb. Gene arrows are drawn according to functional category: transport (dots); regulation, sensing or signaling (stripes); unknown (white). Protein products more abundant in cultures grown with chloromethane (C labeled circles) or with methanol (M labeled circles) are indicated, with black or white symbols used for those proteins observed exclusively or more abundant in one condition, respectively. Proteins homologous to induced genes, or proteins more abundant in a previous study of *M. extorquens* DM4 grown with dichloromethane compared to methanol [16], are indicated with circles labeled by a “D”.

Many of the identified proteins with differential abundance have known or suspected roles in chloromethane utilization and methylotrophy (Table 3). Many of these proteins allowed to define chloromethane-specific clusters encoding proteins more abundant during growth with chloromethane (Fig. 2, Clusters A–F), clusters responding both to chloromethane and methanol (Clusters G–H), and or to methanol only (Clusters I–J). The two-domain methyltransferase/corrinoid binding protein CmuA, the methylcobalamin:H_4_F methyltransferase CmuB, and the formyl-H_4_F hydrolase PurU shown to be essential for chloromethane metabolism in strain CM4, were identified in the chloromethane proteome only (Fig. S1) as expected [6], [9], [10]. Experimental evidence for chloromethane-enhanced expression of a protein involved in cobalamin biosynthesis (precorrin-8X methylmutase CobH2), and of a putative oxidoreductase with FAD/NAD(P)-binding domain encoded by a *paaE*-like gene often associated with *cmu* genes [2], was obtained here for the first time. Overall, only cluster A encoding proteins more abundant during growth with chloromethane (CmuA; CmuB; CobH2; PaaE-like; PurU; Hss2; Table 3) was localized on plasmid pCMU01.

### Proteomic Identification of Stress-related Proteins

Upon dehalogenation, each mole of chloromethane yields one mole of hydrochloric acid with concomitant decrease in pH and increase in chloride concentration [7]. Chloromethane-associated proteins with homologs characterized in *E. coli* for their role in osmoprotection were identified (Proteomic data, Table 3). Among these, protein MdoG may be associated with metabolism of osmoregulated periplasmic glucans [30], the putative dTDP-4-dehydrorhamnose 3,5-epimerase RfbC may be involved in the synthesis of surface polysaccharides [31], and the putative nucleotidase SurE may be associated with survival at high NaCl concentrations, as observed in *E. coli* [32], where the corresponding gene lies within a survival operon conserved in Gram-negative bacteria [33].

Production of reactive oxygen species also seems associated with chloromethane utilization. One representative of each class of catalases known to catalyze disproportionation of hydrogen peroxide (H_2_O_2_) [34] was more abundant in the chloromethane proteome. Mchl_3002 is a putative non-haem manganese-containing catalase. Mchl_3534 is a KatA-like protein, whose gene is found next to a putative H_2_O_2_ activator gene sharing 43% aa Id with *E. coli* OxyR. In *E. coli*, OxyR induces the Suf system (sulfur mobilization [Fe-S] cluster) to combat inactivation of the [Fe-S] Isc assembly system by H_2_O_2_ [35]. Moreover, *E*. *coli* mutants lacking the Suf machinery are hypersensitive to cobalt at high concentrations of 200 µM [36]. In this study, SufS, a selenocysteine lyase homolog, was found more abundant in the chloromethane proteome. Similarly, the CysK cysteine synthase homolog more abundant in chloromethane cultures suggests the probable importance of reactivation systems to maintain chloromethane dehalogenase activity under aerobic conditions, as cysteine is involved in maintaining the catalytic activity and structure of many proteins with [Fe-S] clusters including ferredoxins [37].

Taken together, these data suggest that growth with chloromethane may elicit stress responses, and in particular an oxidative stress response.

## Discussion

This work reports genomic and proteomic data demonstrating that *cmuA* and *cmuB* genes are plasmid-borne, and that plasmid pCMU01drives the previously defined pathway for H_4_F-mediated chloromethane dehalogenation [6]. Specifically, plasmid pCMU01 harbors cognate genes involved in chloromethane-associated H_4_F metabolism not found in other *M. extorquens* genomes (*folC2*, *folD*, *metF2* and *purU*; Table 1) [6], [10].

H_4_F metabolism is likely to be strongly modulated during growth on chloromethane since proteins linked to H_4_F such as CmuA, CmuB, MetF and PurU were exclusively detected during growth with chloromethane (Table 3), whereas proteins associated to methanol oxidation with the metabolic intermediate formaldehyde and the C_1_ carrier H_4_MPT were more abundant during growth with methanol (proteins Fae, Fch and MtdA; Fig. 1).

Here, the interplay of chloromethane and other methylotrophic pathways was evidenced for the first time. Two components of the glycine cleavage complex involved in the conversion of H_4_F and glycine to 5,10-methylene-H_4_F [38], the key C_1_ intermediate for entry in the serine cycle, were either more abundant with chloromethane or with methanol (GcvT and Lpd, respectively; Table 3). This suggests that enzymes implied in central metabolism such as the glycine cleavage complex might be involved in integrating contradictory signals during growth with C_1_ compounds, to fine-tune metabolic conditions required for growth, and to even out variations in available carbon sources.

Our proteomic study also revealed that essential serine cycle enzymes (Sga, HprA and MtkA) were more abundant in methanol-grown cultures (Table 3). These enzymes are encoded by a chromosomal region (Fig. 2, cluster J), highly conserved in *Methylobacterium* [11]. Acetyl-CoA, glyoxylate and NADP^+^ have been demonstrated to decrease binding of QscR, a key regulator of C_1_ metabolism [39] to the *sga* promoter, thereby inhibiting transcription of the major operon of the serine cycle (*sga*- *hpr*-*mtdA*-*fch,* [40]). The higher level of acetyl-CoA synthetase in chloromethane-grown cultures (Table 3) may explain the observed lower abundance of five enzymes encoded by cluster J.

### PaaE-like Oxidoreductase, PntAA, MetF and Acs are Proteins with Predicted Functions for Growth with Chloromethane

Proteomic data provided first experimental evidence for the involvement of four previously undetected proteins, identified here as more abundant during growth with chloromethane, in chloromethane utilization.

The PaaE-like protein encoded by plasmid pCMU01 features a ferredoxin reductase-type FAD binding domain and a 2Fe-2S ferredoxin-type iron-sulfur binding domain. The PaaE-like protein is the only iron-sulfur enzyme more abundant in the chloromethane proteome. This PaaE-like oxidoreductase was suggested to be responsible for the observed methanethiol oxidase activity in the chloromethane-degrading strain, *Aminobacter lissarensis* CC495 [41], [42]. It is also conceivable that the PaaE-like protein acts in the reactivation of the corrinoid cofactor from the inactive Co(II) to the Co(I) form (Fig. 1), as corrinoid-dependent methyltransferases are prone to inactivation by oxidation, and bacteria often require an efficient reactivation system to maintain such proteins in an active form [43], [44]. The implication of PaaE in chloromethane utilization may be linked to the detection of genes associated with the oxidation stress response in chloromethane-grown *M. extorquens* CM4.

The transhydrogenase protein PntAA (Fig. 2 cluster D) couples the transfer of reducing equivalents between NAD(H) and NADP(H) to the translocation of protons across the membrane [45]. Previous transcriptomic and proteomic studies showed that PntAA was up-regulated in succinate- vs methanol-grown cultures of *M. extorquens* AM1 [46], [47], indicating possible differences in energy and reducing equivalent production occurring in *M. extorquens* strains grown on different carbon sources. Here, the higher abundance of the PntAA complex may be the consequence of higher requirements for reducing equivalents coupled to proton extrusion during chloromethane assimilation.

The 5,10-methylene-H_4_F reductase MetF identified in the chloromethane-proteome is the chromosome-encoded protein and not the plasmid-borne protein MetF2 previously shown to be essential for chloromethane utilization [6]. The protein product of *metF2* with a calculated p*I* of 9.5 is at the limit of the pH range studied in our experiments, which may explain why MetF2 was not detected here. In *E*. *coli*, MetF provides one-carbon precursors for methionine synthesis [48] and operates in the opposite direction of the chloromethane degradation pathway. If both MetF and MetF2 homologs share the same metabolites as substrates and products, regulatory processes in the expression of the corresponding genes arising from differences in availability of metabolites may explain the observed increased abundance of MetF with chloromethane. Further experiments are required to clarify the implications of MetF homolgs in chloromethane metabolism.

Identification of the chromosome-encoded acetyl-CoA synthetase Acs as more abundant in the chloromethane proteome was initially surprising. This protein is predicted to catalyze ATP-dependent conversion of acetate to acetyl-CoA (78% aa Id with the characterized *Bradyrhizobium japonicum* Acs enzyme, [49]). Acetyl-CoA is a key metabolite at the interface of C_1_ and multicarbon interconversions involving H_4_MPT-dependent C_1_ subtrate oxidation pathways, the serine cycle, and the ethylmalonyl-CoA pathway essential for growth with methanol [50]. The interconversion of central intermediates such as acetyl-CoA could thus be modulated upon growth with the H_4_F-dependent chloromethane oxidation pathway compared to growth with methanol, resulting in higher abundance of Acs in cells grown with chloromethane.

### Plasmid-encoded BluB2: a Potential Link between H_4_F and AdoCbl Cofactors of Chloromethane Utilization

**Figure 3:**
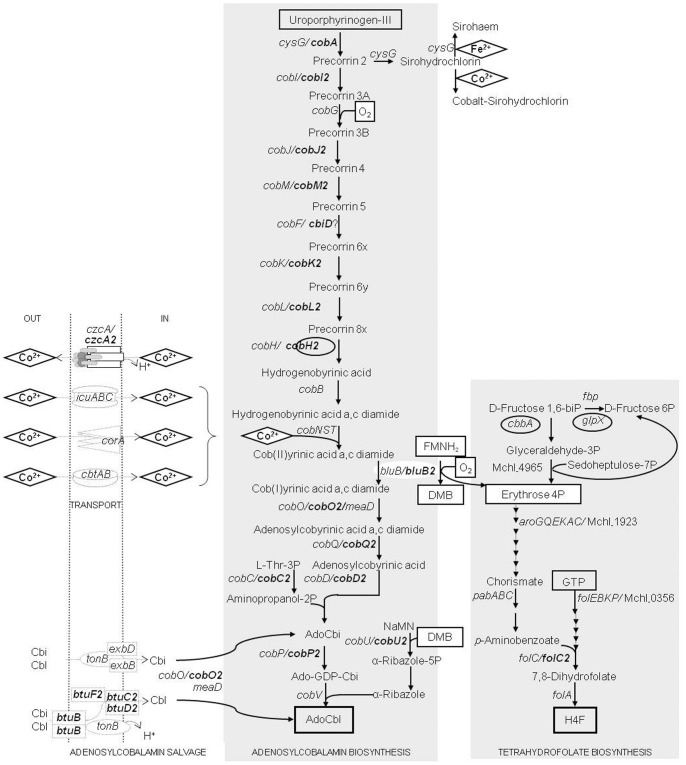
Gene redundancy in the biosynthesis of cofactors required for chloromethane utilization in ***Methylobacterium extorquens*** **CM4.** Cbi, cobinamide; Cbl, cobalamin; Ado, adenosyl; DMB, dimethylbenzimidazole; NaMN, nicotinate mononucleotide. AdoCbl and tetrahydrofolate are essential cofactors of the *cmu* pathway [6], [9]. Transport and enzymatic reactions are shown with dotted and full arrows, respectively. Genes indicated in bold are located on the 380 kb plasmid pCMU01. Circled gene names encode proteins more abundant in chloromethane cultures. AdoCbl can be synthesized *de novo* by an aerobic biosynthesis pathway that incorporates cobalt (diamond), or obtained from a salvage pathway after internalization of preformed Cbi or Cbl. In prokaryotes, the cobalt needed for corrin ring synthesis may be incorporated into cells using the CorA transport system [69], the putative transmembrane proteins CbtA and CbtB [14], the Resistance-Nodulation-Division (RND)-type Co^2+^/Zn^2+^/Cd^2+^ efflux system CzcA [27], or the Icu transporter [70]. The TonB-dependent Btu system imports preformed corrinoid compounds [28]. We hypothesize that BluB-related proteins link AdoCbl and H_4_F *de novo* synthesis.

BluB was demonstrated to catalyze two distinct enzymatic reactions of the AdoCbl biosynthetic pathway: i) conversion of cobinamide to Cbl as a cob(II)yrinic acid a,c-diamide reductase (EC 1.16.8.1) [51]; ii) synthesis of the lower ligand of AdoCbl, dimethylbenzimidazole in Alphaproteobacteria *Sinorhizobium meliloti* [52]and *Rhodospirillum rubrum* [53]. Strain CM4 harbors two *bluB* homologs: *bluB*, conserved in all investigated *M. extorquens* chromosomes, and *bluB2* located on plasmid pCMU01 (38% aa Id between BluB and BluB2; Table 2). BluB2 is highly similar to a characterized enzyme [54] that triggers the oxygen-dependent transformation of reduced flavin mononucleotide (FMNH_2_) in dimethylbenzimidazole and D-erythrose 4-phosphate, a key precursor of chorismate and an intermediate in H_4_F biosynthesis (Fig. 3). Considering the strong level of sequence conservation with proteins of known function (62% aa Id with *S. meliloti* BluB), we speculate that gene *bluB2* may be central for chloromethane assimilation by providing precursors for biosynthesis of essential cofactors of this metabolism.

### Which Role for the Observed Vitamin B_12_-related Gene Redundancy in Chloromethane Metabolism?

Half of the genomes of sequenced prokaryotes that contain homologs of cobalt-utilizing enzymes also possess the AdoCbl biosynthetic pathway, while 90% of the remaining acquire external vitamin B_12_ via the BtuFCD transport system [55]. The genome of *M. extorquens* CM4 contains chromosomal- and plasmid-borne genes for both AdoCbl biosynthesis and corrinoid salvaging via the Btu transporter (Table 2). In addition, plasmid pCMU01 potentially encodes the capacity to remodel exogenous corrinoids, as suggested from the presence of gene *cobO2* encoding the cob(I)yrinic acid a,c-diamide adenosyltransferase enzyme [14] and of gene *cobU2* encoding nicotinate-nucleotide-dimethylbenzimidazole phosphoribosyltransferase (Fig. 3). We speculate that the presence of a variety of corrinoid salvaging pathways, possibly with different substrate affinities and expression profiles (e. g. in response to oxygen, cobalt, vitamin B_12_ or dimethylbenzimidazole availability), may supply *M. extorquens* CM4 with corrinoid coenzyme required for efficient chloromethane dehalogenation in different environments.

Gene duplications are known to correlate with adaptive interactions in prokaryotes by providing competitive advantages for adaptation on specific environmental conditions [56]. Here, *cobA*-encoded S-adenosyl-L-methionine-dependent uroporphyrinogen III methylase (SUMT) may catalyze the transformation of uroporphyrinogen III into precorrin-2 [57] (Fig. 3). SUMT activity may be provided by either CobA and CysG [58], but only CysG is conserved in *Methylobacterium* sequenced strains. Plasmid-borne CobA protein, as a key branch point enzyme in the biosynthesis of modified tetrapyrroles, may favor synthesis of AdoCbl over siroheme driven by chromosome-encoded CysG, control flux to AdoCbl precursor synthesis, and consequently facilitate growth of *M. extorquens* CM4 with chloromethane.

Plasmid pCMU01 encodes a full complement of genes for the corrinoid salvaging pathway, but contains only an incomplete set of genes for the oxygen-dependent AdoCbl *de novo* biosynthesis pathway. Genes *cobB*, *cobF*, *cobG*, *cobN*, *cobS*, *cobT* and *cobV* are missing compared to the canonical pathway characterized in *P. denitrificans* (Fig. 3; Table 2; [14]). However, other yet uncharacterized protein-encoding genes of plasmid pCMU01 may substitute for missing AdoCbl biosynthetic genes. For instance, as previously suggested in *Streptomyces* [59], *cbiD* located next to *cmuBC* encodes a typical oxygen-independent AdoCbl biosynthetic enzyme which, as the missing CobF, features a S-adenosylmethionine binding site. Protein CbiD was reported as a cobalt-precorrin-6A synthase in the anaerobic AdoCbl biosynthesis pathway [60], but its potential role in the corresponding aerobic pathway remains to be investigated.

Suprisingly, out of more than 20 putative vitamin B_12_-related proteins encoded by plasmid pCMU01, only CobH2 was more abundant in the chloromethane proteome (Table 2). A possible explanation for this observation is that vitamin B_12_-related proteins are also required during growth with methanol, e.g. as cobalamin is an essential cofactor for MeaA (ethylmalonyl-CoA mutase) activity in the ethylmalonyl-CoA pathway for glyoxylate regeneration [61].Whether redundant genes encoding vitamin B_12_-related protein in *M. extorquens* CM4 are expressed and functional remains to be evidenced.

Plasmid pCMU01 provides another example of bacterial clustering of genes encoding functional enzymes and cognate genes for cofactor biosynthesis (Fig. 2A). Such a genetic linkage was described for the insect symbiont *Hogdkinia* which has one of the smallest genomes, but dedicates 7% of its proteome to cobalamin synthesis [62]. Similarly, *Lactobacillus reuteri* possesses a metabolic genomic island involved in 3-hydroxypropionaldehyde biosynthesis which associates cobalamin biosynthetic genes and genes of the anaerobic glycerol metabolism [63], most probably reflecting a cobalamin requirement for glycerol dehydratase activity [64], and the polyketide synthesizing bacterium *Streptomyces* sp. DSM4137 has AdoCbl biosynthetic genes adjacent to a putative elaiophylin biosynthetic gene cluster that includes a gene encoding AdoCbl-dependent methylmalonyl-CoA mutase [59]. The genetic linkage of *cmu* and *cob* genes is likely to provide an evolutionary advantage for efficient bacterial growth with chloromethane by the *cmu* pathway.

### Origin and Evolution of Plasmid pCMU01

The mosaic organization of plasmid pCMU01 suggests a history of carbon utilization-related gene acquisition for chloromethane and acetone. Multistep assembly of various genetic elements dedicated to chloromethane utilization in the *cmu* plasmid is supported by several observations: i) A 137 kb segment flanked by IS elements (ISMch8 and ISMch3 of IS*5* and IS*3* family, respectively) contains all hitherto identified chloromethane utilization genes; ii) A 33 kb segment between *cmuBC* and *cmuA* contains remnants of transposase genes (Mchl_5703 and Mchl_5711) and genes involved in corrinoid compound metabolism such as the *cobU2* gene and the putative cobalt heavy metal efflux transporter Czc cluster (Fig. 2A); iii) Atypical genes encoding enzymes relevant to growth on chloromethane such as genes *bluB2*, *cbiA*, and *cobA* are found (Table 1; [65]); iv) The *acxCABR* cluster encoding acetone carboxylase for carbon assimilation is flanked by mobile elements (Table 1 and data not shown); v) 10% of the predicted plasmid CDS are mobile element-related sequences (Table 1); vi) Relics of genes driving plasmid replication (Mchl_5589, 50 residues share 70% aa Id with the N-terminal part of the replication protein RepA, Mchl_5615, 408 residues) suggests that plasmid pCMU01 was assembled by acquisition of parts of different episomes.

### Concluding Comments

Our proteomic analysis showed that the adaptive response of *M. extorquens* CM4 to chloromethane mostly involves functions which are common to *M. extorquens* strains, as observed previously for adaptation of *M. extorquens* DM4 to dichloromethane [16]. Indeed, out of five identified gene clusters responding specifically to chloromethane (Fig. 2), only the catabolic gene cluster essential for growth with chloromethane is encoded by plasmid pCMU01. When these five gene clusters responding specifically to chloromethane in *M. extorquens* CM4 were compared to the seven gene clusters responding specifically to dichloromethane in *M. extorquens* DM4, only one chromosomal gene cluster common to chloromethane-degrading strain CM4 and dichloromethane-degrading strain DM4 was identified (cluster B in Fig. 2; cluster C in [16], suggested to be involved in cell structure). Based on these findings, the adaptive response to growth with chloromethane is clearly quite different from that for growth with dichloromethane, in line with the completely different pathways for metabolism of these two halogenated C_1_ compounds.

On a final note, it is striking that the organization of the *cmu* genes on plasmid pCMU01 of *M. extorquens* CM4 is different from that found in bacteria utilizing the *cmu* pathway known to date [2]. *M. extorquens* CM4 constitutes the first representative strain of the *M. extorquens* species for which a plasmid-encoded carbon utilization function has been clearly established, and its plasmid pCMU01features the only known catabolic gene cluster for the metabolism of halogenated methanes that is not chromosome-borne [4], [11], [16], [66]. The state of plasmid pCMU01 as an autonomous replicating episome may have enabled the efficient acquisition of relevant resources for growth with chloromethane, and the shaping of unique genetic features not observed in other genomes of chloromethane-degrading bacteria.

## Supporting Information

Figure S1 2D-DIGE master gel image of total protein extracts from chloromethane- and methanol-grown *M. extorquens* CM4 labeled with Cy2 (internal standard). Highlighted spots (circles) displayed differential abundance between chloromethane and methanol conditions, and were identified by mass spectrometry. 1, CmuA; 2, CmuB; 3, PurU; 4, PaaE-like oxidoreductase; 5, Fch; 6, Sga; 7, MtdA; 8, putative UspA-like protein; 9, KatA; 10, MetK; 11, Hss; 12, Acs; 13, PntAA; 14, putative endoribonuclease (Mchl_4437) (See Table 3 and Fig. 1 legend).
(TIF)
