# Supplementary figures and images for: The 380 kb pCMU01 Plasmid Encodes Chloromethane Utilization Genes and Redundant Genes for Vitamin B12- and Tetrahydrofolate-Dependent Chloromethane Metabolism in Methylobacterium extorquens CM4: A Proteomic and Bioinformatics Study
Source: PLoS One. 2013 Apr 9;8(4):e56598. doi: 10.1371/journal.pone.0056598 (PMC3621897; doi:10.1371/journal.pone.0056598)

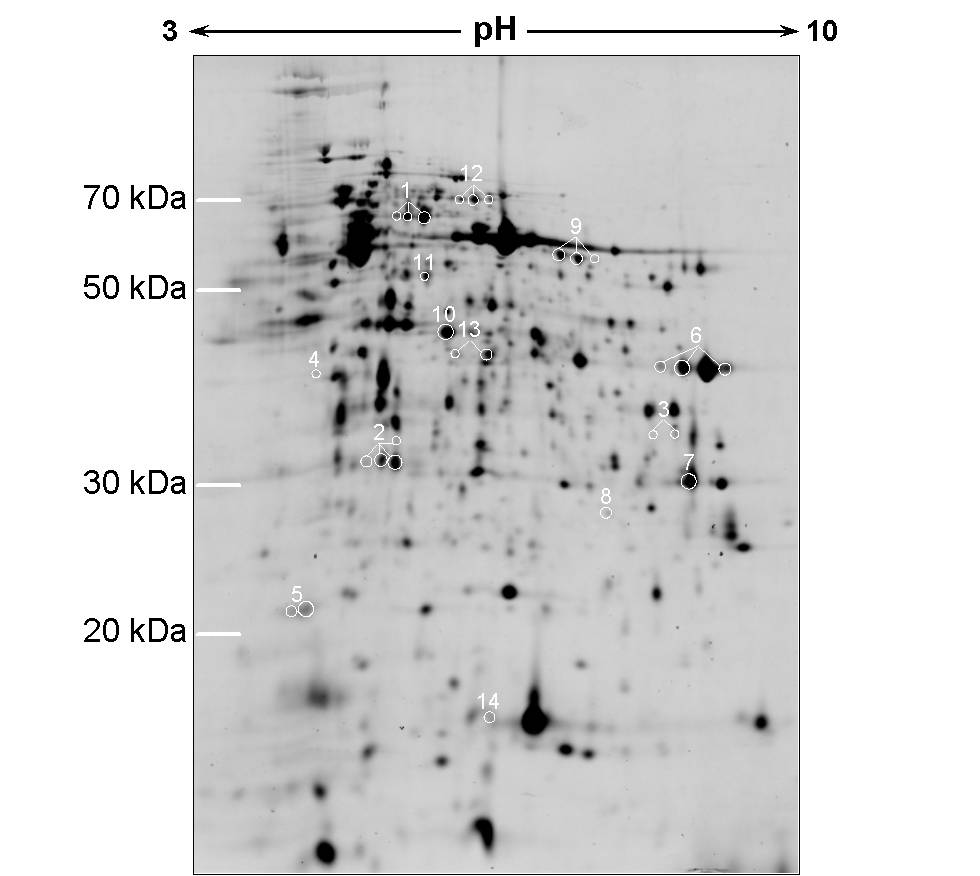

Supplement: Figure S1 — 2D-DIGE master gel image of total protein extracts from chloromethane- and methanol-grown M. extorquens CM4 labeled with Cy2 (internal standard). Highlighted spots (circles) displayed differential abundance between chloromethane and methanol conditions, and were identified by mass spectrometry. 1, CmuA; 2, CmuB; 3, PurU; 4, PaaE-like oxidoreductase; 5, Fch; 6, Sga; 7, MtdA; 8, putative UspA-like protein; 9, KatA; 10, MetK; 11, Hss; 12, Acs; 13, PntAA; 14, putative endoribonuclease (Mchl_4437) (See Table 3 and Fig. 1 legend). (TIF) [file pone.0056598.s001.tif]
